# Policy instruments and self-reported impacts of the adoption of energy saving technologies in the DACH region

**DOI:** 10.1007/s10663-021-09517-6

**Published:** 2021-08-05

**Authors:** Michael Peneder, Spyros Arvanitis, Christian Rammer, Tobias Stucki, Martin Wörter

**Affiliations:** 1 Austrian Institute of Economic Research (WIFO), Vienna, Austria; 2 ETH Zürich, KOF Swiss Economic Institute, Zurich, Switzerland; 3 Center for European Economic Research (ZEW), Mannheim, Germany; 4 Bern University of Applied Sciences, Bern, Switzerland

**Keywords:** Environmental policy, Energy efficiency, Technology adoption, Innovation, Porter hypothesis, Q48, Q55, O13, O25, O33

## Abstract

Using a large sample of enterprises from a survey that was simultaneously conducted in Germany, Austria and Switzerland, we study the self-reported impacts of the adoption of “green” energy saving and related technologies (GETs). Our specific interest is in how different policy instruments associate with energy efficiency, the reduction of $\hbox {CO}_{2}$ emissions, and competitiveness at the firm level. A first set of equations tracks how policy relates to the adoption of green energy technologies in distinct areas such as production, transport, buildings, ICT or renewables. In a second set of equations, we test the perceived impacts of adoption by the managers of the firms. The results confirm a differentiated pattern of varied transmission mechanisms through which policy can affect energy efficiency and $\hbox {CO}_{2}$ emissions, while on average having a neutral impact on the firms’ competitiveness. Further, discarding the conventional dichotomy between incentive-based versus command-and-control type instruments, the results suggest to pursue a comprehensive policy mix, where standards, taxes and subsidies each capitalize on different transmission mechanisms.

## Introduction

The principle of sustainability has become a powerful idea and a key challenge for societies to cope with. In 2015 the United Nations (UN) launched its Agenda for Sustainable Development with an aim to pave the way for “prosperity and opportunity for all on a healthy planet.”^1^ The UN further elaborated its ambitious objectives by defining 17 *Sustainable Development Goals* (SDGs), among which SDG8 occupies a central position for simultaneously promoting sustainable and inclusive economic growth and employment. Among others, Stern and Stiglitz (2021) highlight the strong connection between these dimensions.^2^ Similarly, Mealy and Hepburn (2021, 397) point out that “traditional industrial routes out of poverty are dangerously emissions-intensive” and discuss the common features of transformational change towards the UN climate and development goals. 2021 further stress the importance of new green technologies and products in order to achieve a sustainable growth path. Finally, Stern and Stiglitz (2021, p. 19) also find reason for optimism when it comes to the role of new technology: “The fossil-fuel economy was borne of innovation, and it could as well die as a result of innovation.”

This paper investigates how public policies associate with the adoption of new green energy technologies (GETs) and the self-reported impacts on competitiveness in a large sample of individual companies. Within the far-reaching agenda of the SDGs, it aims to contribute in particular at the intersection of energy efficiency, technological change and climate change.

But do we really need policy to interfere with the energy efficiency of private businesses? From a conventional economics perspective, there are valid reasons to answer in the negative. Higher efficiency implies lower cost to the individual enterprise, thus providing private incentives to adopt new GETs as long as the savings match the cost of investment. Also for most non-renewable energy sources property rights are well established. If consumption is excludable, prices reflect rents from scarcity and the rate of exhaustion should be welfare-efficient (Hotelling 1931). In contrast, Solow (1992) defined sustainability as an ethical norm of inter-generational equity: to ensure that future generations can be as well off as we are. The distant future, however, is not well represented in the market, and opinion surveys have revealed comparatively low concerns in the overall population about the natural environment.^3^ As a consequence, sustainability is not adequately covered by private decisions and the conventional rationale of allocative efficiency. Hence, robust concerns for sustainability arise with regard to the emission of green house gases and the consequent perils of climate change, where the sheer scope of the problem dwarfs many of the economist’s standard analytic premises. When assessing the benefits and costs of public intervention, the very long time horizon, uncertainty, nonlinear impacts and the related risk of irreversible, catastrophic events largely obliterate the use of expected values or market-based discount rates, instead calling for a general conservationist bias.^4^

Most rationales of public intervention originate in the so called *double externality* problem, which points at the simultaneous occurrence of negative spillovers from pollution and exhaustible resources in addition to the positive spillovers from innovation and the adoption of “greener” technologies.^5^ Yet in the presence of increasing returns these ecological problems are likely to be amplified by path-dependency and lock-in effects. Therefore, the conventional focus on cost-efficiency and the correction of relative prices alone won’t suffice to change trajectories.^6^

According to Frankel (2004), societies face three distinct paths of transformation: One is to address the scale of operations—that is, pursuing less or no growth with all its consequences of foregone real income and distributional conflicts, especially between developed and developing regions. Even if cushioned by far reaching measures in terms of the redistribution of global incomes, the negative trade-off between such a strategy of *de-growth* and the UN’s SDG8 are obvious. A second path is to change the *composition* of activities. At high per capita income, environmental efficiency tends to improve, because of structural change in favour of services and preferences for a clean environment.^7^ While this contributes to the decoupling of the growth of emissions relative to that of output, growth is nevertheless likely to further increase the cumulative stock of pollutants. *Innovation* and technological change open a third path, which enhances structural change but also reduces the emissions of given activities. At a fundamental theoretical level, it corresponds to the tendency of dissipative systems to either increase the access to free energy or to raise the efficiency in its use.^8^ However, whether innovation and structural change ultimately can be sufficient to decouple material well-being from resource throughput will depend on the aggregate rates of growth in resource efficiency relative to that of labour productivity and of the population (Bretschger 2020; Hahnel 2021). In more practical terms, Nordhaus (2019, p. 2013) emphasises that technological change lowers the cost of reducing $\hbox {CO}_2$ emissions and provides for a “last refuge” if other policies fail.

The focus of our research is on such technological change. But unlike the growing literature on own environmental innovations, which uses patents, R&D expenditures or other relatively well-available measures from common innovation surveys as dependent variables, we are interested in the *adoption* and perceived impacts of the new technologies. Our central concern is how different policy instruments such as standards and negotiated agreements, taxes or subsidies are related to the adoption of GETs by the companies and thus to their self-reported environmental impacts and competitive position in the market. For this purpose, we had to create a new enterprise survey, which we then conducted simultaneously in Germany, Austria and Switzerland (the ‘DACH region’).

There are obvious similarities in how the three countries deploy the policy instruments mentioned above. For instance, in the field of energy policy guaranteed feed-in tariffs for electricity from renewable sources exist in the entire region. They intend to foster investments in the production of renewable energy by reducing the uncertainty about future demand and prices, but also imply that final users must bear higher electricity prices. Similarly, all three countries levy various carbon taxes, e.g. on fossil fuels, and participate in trading systems for carbon emissions. Finally, in innovation policy the growing concerns about climate change tend to shift funding priorities in favour of GETs.

But each country has its distinct history, institutional setting and policy framework. For example, during the past decades *Germany* has put much emphasis on the promotion of renewable energy,^9^ which is expected to substitute for nuclear power after its planned fade out. Germany was the first of the three countries to introduce guaranteed feed-in tariffs^10^ for electricity from renewable sources already in 1991. This contributed greatly to the remarkable increase of the share of wind and solar power in total electricity production.^11^ Another policy that led to higher energy prices was the ecological tax reform in 1999, which included the introduction of a new electricity tax. In addition, the petroleum tax was redesigned to provide better incentives for the purchase of energy-efficient vehicles. A further example is the CO2 Building Rehabilitation Programme aimed at incresing the incentives for energy-efficient building refurbishments. The German Federal Government is also running several R&D programmes that foster the development of energy-saving technologies. Generally, there are no large-scale voluntary agreements on energy saving or energy efficiency on a cross-industry level, though individual industries, such as the chemical industry or the automotive sector, have been following such an approach.

In *Austria* the relative abundance of hydraulic power has provided a comparative advantage to energy intensive industries, such as metal processing or cellulose and paper, which still produce a comparatively high shares of manufacturing output. Another particular characteristic is Austria’s ban on nuclear energy, which was enacted after a public referendum in 1979. Energy policy thus always ranked relatively high on the public agenda, while energy intensive producers enjoy considerable political leverage.^12^ In 2015 the new *Energy Efficiency Law* became the focal point of the country’s regulatory approach in this area. After adopting EU guidelines, energy suppliers (except small ones) must prove concrete measures to achieve annual efficiency gains of 0.6% relative to their previous year’s total energy sales. These efficiency gains may originate either from their own operations or from their customers and depend on the ratio of energy inputs to output (i.e. not on total energy use).^13^ In addition, the law commands large companies either to install a proper energy management system (EMS), or to have an energy audit every four years.

Compared to Germany and Austria, *Swiss* policy has generally been more reluctant to intervene in favour of GETs. The Swiss framework is mainly characterized by market incentives and voluntary agreements.^14^ Switzerland nevertheless has some important targeted policies, though these were often introduced much later than in the two other countries. For example, in 2008 the first commitment period of the Kyoto protocol initiated a carbon tax and a (still rather narrow) emission trading system started in 2013. Similar to other rules applied in the European Union, there are regulations of, e.g., emissions of passenger cars, or the labeling, promotion and installation of renewable energy plants. Public subsidies are available in form of a technology fund to promote innovations for reducing greenhouse gas emissions and the consumption of resources, supporting also the use of renewable energy and the increase of energy efficiency. There are also subsidies for basic research and applied R&D in the form of pilot plants related to GETs.

After these introductory remarks, Sect. 2 introduces the main heuristics and hypotheses, whereas Sect. 3 presents the data and descriptive statistics. Section 4 explains the econometric model and results. Section 5 summarizes and concludes. Finally, we provide a list of abbreviations, references, and an Annex with supplementary tables.

## Heuristics and hypotheses

As stated in the introduction, the core question of this research is how environmental policies relate to the perceived impacts on the competitiveness, energy efficiency and $\hbox {CO}_2$ emissions of individual firms in Germany, Austria and Switzerland. By aiming to induce firms to adopt certain practices and technologies to achieve desired ecological impacts, the transmission mechanism and hence the nature of interventions must be indirect. Consequently, we separate the general problem into three consecutive questions: First, whether policy associates with more adoption of new energy efficient technologies and higher shares of GETs in total investments. Second, whether firms perceive that these activities actually lead to the desired ecological effect of boosting energy efficiency and reducing $\hbox {CO}_{2}$ emission. Third, and closely related to the second question, policy is also interested in the opportunity cost of interventions—i.e. whether firms perceive the induced actions to have negative, neutral or positive impacts on their competitiveness.

The comprehensive nature of the enterprise survey (see Sect. 3.1) allows us to test the various channels of transmission in two broad sets of equations. The first set is comprised of nine equations that explain the extensive margin of adoption for various areas of technology and the overall intensive margin by means of the vector of inducement factors and the general control variables.^15^ The second set of three equations turns to the perceived impacts of adoption on the firm’s energy efficiency, $\hbox {CO}_{2}$ emissions and competitiveness. Both types of equations can only produce *positive* statements, which refer to the importance of actual policies and impacts of the firms in our sample, i.e. as observed for the DACH region during that period. A low or insignificant coefficient of any instrument may thus be due either to the insufficient scope of (an otherwise effective) intervention, its inefficient implementation, or a bad choice of policies. Similarly, significant coefficients can only indicate that a certain policy appears in principle to be effective, demonstrating a positive statistical association for our sample of firms in the DACH region.

**Fig. 1:**
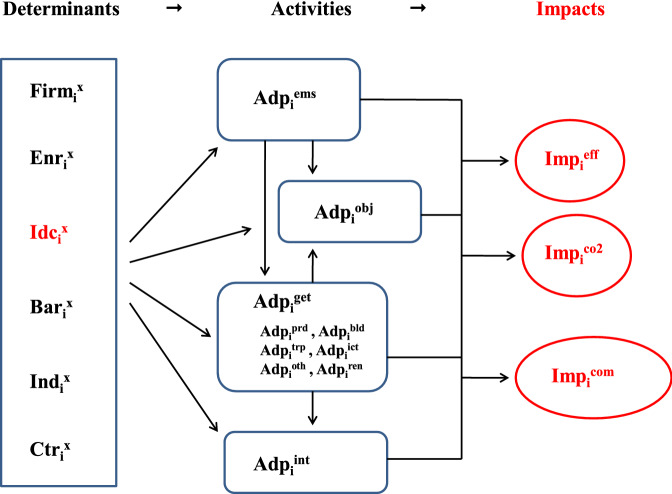
Basic organisation of the estimated equations. NB: For firm *i* and variable *x*: Enr = energy-related factors, Idc = inducement factors, Bar = Barriers, Ind = industry, Ctr = country, Adp = Adoption, Imp = Impacts, ems = energy management system, obj = objectives, get = green energy technology, prd = production, trp = transport, bld = buildings, ict = ICT, oth = other, ren = renewable energy, eff = energy efficiency, co2 = $\hbox {CO}_2$, com = competitiveness. See Tables 1 and 2 for a comprehensive description of the variables

In order to keep track of the many equations and variables, Fig. 1 provides a simple representation of the heuristic model. Despite its apparent complexity, the model aims for a straightforward chain of relationships, which goes from policy to adoption and then from adoption to ecological and economic impacts. Though we cannot rule out significant problems of endogeneity, there are no plain reasons to suspect them, except if in the longer run past experiences shape current expectations with respect to effects. Given the limitation of the purely cross-sectional data at hand, a credible structure of exogenous effects is essential to approaching a meaningful interpretation of the data.

Another means of staying focused on the guiding questions of our research is to deliberately expatiate the particular hypotheses for the core relationships that we aim to test. Though some may appear obvious if taken on their own, stating them explicitly highlights their relevance to the overall transmission from policy to the ecological impacts and competitiveness. To begin with, three hypotheses address the expected association of the respective *policy inducement* factors with adoption:

- **Extensive margin** (*H*1): Policy *x* associates positively with the firms propensity to adopt new GETs. It must be rejected, if the policy either shows no significant coefficient or associates with a significant decrease in the probability of adopting new GETs.
- **Intensive margin** (*H*2): Policy *x* associates with a higher share of expenditures for new GETs in total investments. It must be rejected, if the policy associates with a decrease or does not significantly relate to the intensive margin of adoption.
- **Heterogenous** effects of different policy instruments (*H*3): The statistical association of different policies varies according to particular aspects of adoption, such as the primary motivation, the introduction of EMS, extensive and intensive margins, or different areas of technology.

The latter hypothesis is explorative, since little is known about it from the literature. The theoretic model and numeric simulations of Acemoglu et al. (2012, 2016) support the idea that a combination of policies is preferable to choosing only one instrument. Moreover, for a given technical specification standards can be expected to primarily influence the extensive margin of adoption due to their mandatory and discrete nature. In contrast, subsidies and taxes might have a greater impact on the intensive margin of adoption as they continue to vary with the further effort invested either below or above a technical threshold. Finally, standards are the most direct means of how policy can shape the firms’ selection environment and one may therefore expect them to be the most commonly relevant factor in the adoption of new GETs.

A further two hypotheses address the self-reported *ecological impacts* of adoption:

- **Perceived impact on energy efficiency** (*H*4): The adoption of GETs tends to increase the self-reported energy efficiency of firms. It must be rejected, if adoption has a significant negative association or does not significantly relate to energy efficiency.
- **Perceived impact on** $\hbox {CO}_{2}$ **emissions** (*H*5): The adoption of GETs tends to reduce the self-reported $\hbox {CO}_{2}$ emissions of firms. It must be rejected, if adoption has a significant positive association or does not significantly relate to $\hbox {CO}_{2}$ emissions.

Turning to the self-reported *impacts on competitiveness*, contemporary concepts at the aggregate level emphasize the positive contribution of cleaner production to a society’s overall standards of living. However, these social benefits are largely external to the individual firm, which bears the private cost of abatement and compliance to environmental regulations (Pasurka 2008).^16^ The immediate impact of regulation is thus to add or tighten constraints on a firm’s set of choices (Palmer et al. 1995; Berestycki and Dechezleprêtre 2020), which inflicts additional cost to the enterprise and depresses its competitiveness, if rival enterprises face fewer constraints. This argument leads us to the first of three competing hypotheses:

- **Conventional trade-off hypothesis** (*H*6*a*): The adoption of new GETs associates with a self-reported decrease in the competitiveness of firms. It must be rejected if it associates significantly with its increase or does not significantly relate to it.

In contrast, generalising the insights from a rich repository of case studies, Porter (1990) and Porter and van der Linde (1995) argue to relax the conventional trade-off between competitiveness and environmental policy. They demonstrate how well-designed, preferably incentive-based regulations can alert individual companies, which are often captive to myopic optimisation within a given market environment, to better anticipate long-run trends in demand or international regulations. For a given location, a stricter regulatory environment can thus induce early innovations and first-mover advantages with regard to environmentally friendly products and processes.^17^

- **Porter hypothesis** (*H*6*b*): The introduction of new GETs associates with a self-reported increase in the competitiveness of firms. It must be rejected if it associates significantly with its decrease or does not significantly relate to it.

The Porter hypothesis has triggered much controversy and has provided a fruitful platform for further research. It has offered stronger theoretical explanations^18^ and robust empirical support for a weaker restatement, which predicts a positive impact of environmental regulation on innovation (Jaffe and Palmer 1997). Evidence of its initial strong prediction of a positive impact on competitiveness is, however, mixed.

One likely explanation is that environmental regulations apply uniformly to a given firm population, whereas the induced innovation races tend to produce skewed returns (Popp 2005). Typically, the winner takes all or at least a large chunk of the innovation rent, sharing the remainder with firms that rapidly adopt the new technology. Consistent with its initial case study approach, the Porter hypothesis should therefore apply to the winners of an innovation race and some fast followers, but not to an entire cross-section of enterprises. Furthermore, in the case of technology adoption, the needs and incentives are similar for firms operating within the same market, leaving little scope for differential impacts. Finally, considering the special nature of GETs, where the increased energy efficiency compensates for (at least part of the policy induced) expenditures on adoption, our preferred hypothesis predicts a neutral impact on the current cross-section of firms:

- **Neutrality hypothesis** (*H*6*c*): The adoption of new GETs does not significantly associate with the competitiveness of the average enterprise in a cross-section of firms. It must be rejected if it significantly associates with either an increase or decrease of the self-reported competitiveness of the average firm.

## Data

### The enterprise survey

The data used in the analysis originate from a comprehensive enterprise survey conducted in Germany, Austria and Switzerland. The survey focused on the creation and adoption of new energy saving and related technologies. It was jointly developed and simultaneously launched in the summer of 2015 by the ETH Zürich, the Center of European Studies (ZEW) and the Austrian Institute of Economic Research (WIFO). In total, the gross sample amounted to 19,254 firms. The firm samples are comprised of manufacturing, construction and services, except for the energy sector and state-related services, such as public administration, education or health. They were built from a stratified random sample, with stratification applied to two-digit industries and three industry-specific classes of firm size but full coverage of large firms. Comprehensive recall actions in all three countries ensured a sufficient number of respondents covering all industries and size classes according to the sampling scheme. Only for Austria some cells could not be filled separately and were therefore merged with a neighbouring cell. The net sample of valid responses includes 4,634 firms, 49% of them in Germany, 39% in Switzerland and 12% in Austria.

The questionnaire asked about basic firm characteristics, general energy-related characteristics as well as inducement factors and barriers to the adoption or own innovation of GETs. Innovation related questions, however, were only directed at a subgroup of manufacturing sectors (excluding food, textiles and clothing, printing, pharmaceuticals, and other miscallenous manufacturing). Consequently, the number of observations considerably declines for all estimations where own innovation is included among the regressors.

The identification of the relevance of different energy policies at the firm level is hardly possible through data from public sources. We hence asked managers to rate the relevance to their business on a three-point Likert scale.^19^ The big advantage is that we can thus cover all types of policies on the same scale. However, self-reported assessments require considerable caution in the interpretation of results (Bertrand and Mullainathan 2009). One obvious problem is their limited comparability across firms, which can add much noise to the data. More importantly, systematic distortions can arise from endogeneity, if the subjective assessments are correlated with unobserved firm specific factors. Our strategy here is to apply a large set of control variables. Obviously, the opportunity to design a new survey specifically for the purpose of this research was extremely helpful to mitigate the risks of such a bias from omitted variables.

Developing our own survey also allowed us to reduce the risk of a common response bias. Avoiding a frequent problem of enterprise surveys, we deliberately asked all firms about the relevance of the various policy related factors, irrespective of whether they had actually adopted new GETs or not. Thus we can also establish the relevance of the inducement factors when policies target different firms, originate from multiple territorial levels, or are subject to imperfect monitoring and enforcement.^20^

### The variables

The variables are organised along the three dimensions of (i) determinants, (ii) activities and (iii) impacts. Among the independent *determinants*, we distinguish between general firm characteristics (*Firm*), specific energy-related factors (*Enr*), inducement factors (*Idc*), barriers to adoption (*Bar*), fixed effects for the industry (*Ind*) and the country (*Ctr*) in which the responding firm *i* is located. Tables 1 and 2 provide the labels and definitions for each variable used in the analysis.

**Table 1:** Variables, labels and definitions: (i) determinants

Variable	Label	Description
Determinants		
$Firm_i^{grp}$	Group	Dummy whether the firm is part of a group of companies
$Firm_i^{age}$	Age	Survey year minus the firm’s start of operations
$Firm_i^{sze}$	Size	Three classes by number of employees (< 50/51 - 250/> 250)
$Firm_i^{com}$	Competition	Number of competitors for the firm’s principal product
$Firm_i^{exp}$	Exports	Dummy whether the firm exports
$Firm_i^{ino}$	Innovation (GET)	Dummy whether the firm reported own innovations in GETs
$Enr_i^{cos}$	Energy cost	Share of expenditures on energy inputs in total sales (2012)
$Enr_i^{pri}$	Energy prices	Importance of high and volatile energy prices (2012 to 2014; not/somewhat/highly relevant)
$Enr_i^{sht}$	Energy shortages	Importance of (fears of) energy shortages (2012 to 2014; see above)
$Enr_i^{mix}$	Energy mix	Change in energy mix (none/change with/without cost reduction)
$Idc_i^{dem}$	Demand	Importance of customer demand for energy efficient goods and services (2012 to 2014; see above)
$Idc_i^{pfu}$	Public funding	Importance of energy-related public funding (see above)
$Idc_i^{tax}$	Taxes	Importance of energy-related taxes and duties (see above)
$Idc_i^{reg}$	Regulation	Importance of energy-related regulations (see above)
$Idc_i^{std}$	Standards	Importance of energy-related standards or negotiated environmental agreements (see above)
$Bar_i^{frc}$	Polit. framework	Impeded by political framework (2012 to 2014; four scales, “no “to “very relevant“)
$Bar_i^{inc}$	Techn. incompat.	Whether technological incompatibilities impeded adoption (see above)
$Bar_i^{imm}$	Immature techn.	Whether immature technology impeded adoption (see above)
$Bar_i^{prm}$	Permits	Whether costly procedures impeded adoption (see above)
$Bar_i^{amr}$	Amortisation	Whether long periods for amortisation impeded adoption (see above)
$Bar_i^{fin}$	Finance	Whether lack of finance impeded adoption (see above)
$Ctr_i^{at,ch}$	Country	Two dummies for firms located in Austria and Switzerland (Germany is comparison group)
$Ind_i^{nace}$	Industry	Sector dummies at the level of NACE 2-digits

**Table 2:** Variables, labels and definitions: (ii) activities and impacts

Variable	Label	Description
Activities		
$Adp_i^{ems}$	EMS	Dummy for certified management system or audits related to energy
$Adp_i^{obj}$	Objectives	Whether adoption aimed for energy efficiency (secondary effect/both/primary objective)
$Adp_i^{get}$	Extensive margin	Adoption of new GETs in any of the following areas:
$Adp_i^{prd}$	... Production	Adoption related to production
$Adp_i^{bld}$	... Buildings	Adoption related to construction & buildings
$Adp_i^{trp}$	... Transport	Adoption related to transport
$Adp_i^{ict}$	... ICT	Adoption related to ICTs
$Adp_i^{oth}$	... Other	Adoption related to other areas
$Adp_i^{ren}$	... Renewable energy	Adoption related to the use of renewable sources
$Adp_i^{int}$	Intensive margin	Share of expenditures on new GETs in total investments
Impacts		
$Imp_i^{eff}$	Energy efficiency	Energy consumption per unit or process (not improved/can’t say/improved/much improved)
$Imp_i^{co2}$	$\hbox {CO}_{2}$ emissions	$\hbox {CO}_{2}$ emissions per unit or process (see above)
$Imp_i^{cmp}$	Competitiveness	Competitive position on the market (worsened/didn’t change/improved/much improved)

Among the inducement factors, we distinguish five types: (a) energy-related taxes and duties; (b) subsidies for developing and adopting green energy technologies; (c) standards and negotiated agreements; (d) other regulations on energy use, such as emissions caps and certificates,^21^ and (e) customer demand for energy-efficient products or products.

Among activities, we distinguish between the adoption ($Adp_i^x$) of energy-related management systems or other measures for the regular audit of energy use and environmental impact ($x=ems$) on the one hand, and green energy technologies ($x=get$) on the other. We further separate the latter into those for production ($x=prd$), construction and building ($x=bld$), transportation ($x=trp$), information and communication technologies ($x=ict$), other GETs ($x=oth$) or renewable energy sources ($x=ren$). These dichotomous variables capture the specific *extensive* margin of adoption by technology field. In contrast, we measure the overall *intensive margin* by the share of total expenditures on the adoption of any GETs in total investments of the firm ($Adp_i^{int}$). In addition, we aimed to control for the genuine motivation of adoption, asking whether an increase in energy efficiency or reduction of $\hbox {CO}_{2}$ emissions was a primary objective or secondary effect of the investment ($x=Adp_i^{obj}$).

Finally, the impact variables $Imp_i^x$ bring in the self-reported assessment of the respondents, whether and to what degree the adoption of new GETs has improved performance with regard to energy consumption per unit or process ($x=eff$), CO2 emissions per unit or process ($x=co2$), and whether the competitive position on the market has worsened, not been affected, improved or much improved as a consequence of the adoption of GETs ($x=com$).

### Descriptive statistics

The basic descriptive statistics for each variable are summarised in Table 3.^22^ Among the 4,634 valid observations of the enterprise survey, the firms in the sample have on average 269 employees. The median is 38 employees. About half of the firms belong to industrial production^23^ and half to services (including construction). About 27% of the firms have introduced a certified EMS and 47% have introduced GETs. Among these, a majority of 1452 firms has adopted new GETs in the area of construction and buildings. 978 adopters did so in the field of information and communication technologies (ICTs), closely followed by 911 firms introducing them in the field of production. 645 and 456 firms reported new GETs with regard to transport and renewable energy. Only a small fraction referred to adoption in other fields, such as the cogeneration of heat and power.^24^ The pairwise correlation of policy factors with the introduction of EMS and GETs is strongest for standards, followed by public funding. In the case of GETS, the association is similar for taxes but weakest for regulation, whereas the reverse applies to EMS (Table 4).

**Table 3:** Basic descriptive statistics

Variable	Observ.	Mean	StDev	Min	Max
$Firm_i^{grp}$	4190	0.320	0.467	0	1
$Firm_i^{age}$	4537	46.585	42.721	0	514
$Firm_i^{sze}$	4634	1.568	0.696	1	3
$Firm_i^{com}$	4292	2.598	1.509	1	5
$Firm_i^{exp}$	4376	0.510	0.500	0	1
$Firm_i^{ino}$	2186	0.153	0.360	0	1
$Enr_i^{cos}$	3705	2.908	7.093	0	280
$Enr_i^{pri}$	4547	1.770	0.737	1	3
$Enr_i^{sht}$	4547	1.254	0.546	1	3
$Enr_i^{mix}$	4529	0.300	0.682	0	2
$Idc_i^{dem}$	4634	0.324	0.600	0	2
$Idc_i^{pfu}$	4634	0.494	0.697	0	2
$Idc_i^{tax}$	4547	1.689	0.739	0	2
$Idc_i^{reg}$	4634	0.465	0.681	0	2
$Idc_i^{std}$	4634	0.379	0.629	0	2
$Bar_i^{frc}$	4344	1.646	0.965	1	4
$Bar_i^{inc}$	4344	1.459	0.821	1	4
$Bar_i^{imm}$	4344	1.591	0.895	1	4
$Bar_i^{prm}$	4344	1.511	0.866	1	4
$Bar_i^{amr}$	4344	1.838	1.096	1	4
$Bar_i^{fin}$	4344	1.523	0.903	1	4
$Ctr_i^{at,ch}$	4634	na	na	0	1
$Ind_i^{nace}$	4634	na	na	0	1
$Adp_i^{ems}$	4634	0.271	0.445	0	1
$Adp_i^{obj}$	2051	1.806	0.793	1	3
$Adp_i^{get}$	4634	0.468	0.499	0	1
$Adp_i^{prd}$	4634	0.202	0.402	0	1
$Adp_i^{bld}$	4634	0.327	0.469	0	1
$Adp_i^{trp}$	4634	0.143	0.350	0	1
$Adp_i^{ict}$	4634	0.220	0.415	0	1
$Adp_i^{oth}$	4634	0.022	0.147	0	1
$Adp_i^{ren}$	4634	0.103	0.304	0	1
$Adp_i^{int}$	4634	5.360	13.322	0	100
$Imp_i^{eff}$	2053	2.849	0.959	1	4
$Imp_i^{co2}$	2011	2.585	0.973	1	4
$Imp_i^{cmp}$	2054	1.516	1.082	0	3

**Table 4:** Pairwise correlation of policy with EMS and GET by technology

	Public funding	Taxes	Regulation	Standards
	*Coefficients of correlation*			
EMS	0.273	0.245	0.269	0.280
GET total	0.205	0.204	0.194	0.217
Production	0.245	0.263	0.244	0.244
Buildings	0.197	0.188	0.193	0.223
Transport	0.128	0.100	0.131	0.152
ICT	0.114	0.079	0.107	0.126
Other	0.072	0.102	0.065	0.054
Renewables	0.095	0.081	0.090	0.108

Among all GET-adopting firms, about 24% claim that energy efficiency was a primary motive. 43% of the respondents consider it a secondary impact and 33% report that both applied (Table 5). Less than 10% of adopting firms report that it did not improve their energy consumption per unit or process, and about 25% can’t tell. Conversely, 36% report that their energy efficiency has improved and 30% that it has much improved due to the adoption. For $\hbox {CO}_{2}$ emissions the perceived impacts are similar but somewhat lower. Finally, with regard to the impacts on the firm’s competitiveness, 23% say that it was negative, whereas 25% report no change. This leaves only a small majority to those firms that actually experienced a positive effect. Moreover, the impacts of new GETs on the competitiveness of the firm show little variation between different technological areas.

**Table 5:** Objectives and impacts by area of GET adoption

	Production	Buildings	Transport	ICT	Other	Renewables	Total
	*Share of adopting firms in %*						
Primary or secondary objective							
Secondary	36.70	38.39	35.19	46.37	19.39	23.09	43.00
Both	38.13	34.73	39.35	34.29	40.82	37.69	33.40
Primary	25.16	26.88	25.46	19.34	39.80	39.22	23.60
*Total*	*100.0*	*100.0*	*100.0*	*100.0*	*100.0*	*100.0*	*100.0*
Impact on energy efficiency							
Not improved	7.24	8.26	9.46	9.10	8.25	9.87	9.99
Can’t say	16.90	22.59	20.62	25.97	22.68	22.59	24.65
Improved	41.27	36.36	37.36	37.12	46.39	33.99	35.80
Much improved	34.58	32.78	32.56	27.81	22.68	33.55	29.57
*Total*	*100.0*	*100.0*	*100.0*	*100.0*	*100.0*	*100.0*	*100.0*
Impact on $\hbox {CO}_{2}$ emission							
Not improved	10.33	11.28	9.36	12.68	6.32	9.78	12.63
Can’t say	31.65	37.09	31.51	38.88	36.84	35.33	38.79
Improved	31.43	27.57	29.02	25.78	33.68	27.11	26.01
Much improved	26.60	24.05	30.11	22.66	23.16	27.78	22.58
*Total*	*100.0*	*100.0*	*100.0*	*100.0*	*100.0*	*100.0*	*100.0*
Impact on competitiveness							
Worsened	24.86	23.61	20.87	23.58	26.80	21.15	23.22
No change	28.38	24.43	26.74	25.61	48.45	23.35	24.83
Improved	22.66	27.87	27.51	30.08	24.74	29.52	29.11
Much improved	24.09	24.09	24.88	20.73	0.00	25.99	22.83
*Total*	*100.0*	*100.0*	*100.0*	*100.0*	*100.0*	*100.0*	*100.0*

## Econometric analysis

The focus of the analysis is on how different policy instruments associate with (i) energy efficiency, (ii) the reduction of $\hbox {CO}_{2}$ emissions, and (iii) competitiveness at the firm level. We thereby distinguish between two mechanisms: First, the *adoption* equations explain how various determinants, including policy, relate to the adoption of new GETs. Second, the *impact* equations test whether the adoption of GETs associates significantly with improved ecological impacts and how it relates to the firm’s competitiveness.

### Specifications

For the econometric specification, we again start with the impact of policy on adoption. On the left side, we find the dependent variables for each of the *n* equations. On the right side, the first vector gives the constant intercept $\alpha$, the second vector the *k* common independent variables, depicted by the coefficients $\beta _n^k$, and finally the error terms $\upsilon _n$.

1 $$\begin{aligned} \begin{pmatrix} Adp_i^{ems} &{}\\ Adp_i^{get} &{}\\ Adp_i^{int} &{}\\ Adp_i^{obj} &{}\\ \end{pmatrix} = \begin{pmatrix} \alpha _1 + &{} \beta _1^k X_i^k &{} + \upsilon _1 \\ \alpha _2 + &{} \beta _2^k X_i^k &{} + \upsilon _2 \\ \alpha _3 + &{} \beta _3^k X_i^k &{} + \upsilon _3 \\ \alpha _4 + &{} \beta _4^k X_i^k &{} + \upsilon _4 \end{pmatrix} \end{aligned}$$

The metric of the dependent variables determines the choice of the appropriate method of estimation. For the dichotomous extensive margins of adoption $Adp_i^{ems}$ and $Adp_i^{get}$ we use *probit* regressions, which apply the maximum likelihood principle to cumulative normal distributions. The coefficients tell the impact of the independent variables on the respective response probabilities. Analogously, we apply an *ordered probit* regression to fit $Adp_i^{obj}$. The dependent variable is again discrete, but has three possible ordinal outcomes. Finally, the continuous nature of the intensive margin $Adp_i^{int}$ allows for estimation by *ordinary least squares* (OLS). Alternative methods (e.g., *logit*, *multinomial logit*, or the *linear probability* model for discrete variables) are used to test the robustness of the empirical findings.

For the individual technology fields, we use a *multivariate probit* model, which applies the method of simulated maximum likelihood (SML) to jointly fit the five different binary choices of adoption covered by the survey:^25^

2 $$\begin{aligned} \begin{pmatrix} Adp_i^{prd} &{}\\ Adp_i^{bld} &{}\\ Adp_i^{trp} &{}\\ Adp_i^{oth} &{}\\ Adp_i^{ren} &{}\\ \end{pmatrix} = \begin{pmatrix} \alpha _5 + &{} \beta _5^k X_i^k &{} + \upsilon _5 \\ \alpha _6 + &{} \beta _6^k X_i^k &{} + \upsilon _6 \\ \alpha _7 + &{} \beta _7^k X_i^k &{} + \upsilon _7 \\ \alpha _8 + &{} \beta _8^k X_i^k &{} + \upsilon _8 \\ \alpha _9 + &{} \beta _9^k X_i^k &{} + \upsilon _9 \end{pmatrix} \end{aligned}$$

Reflecting the different dimensions of the explanatory variables in Eqs. (1) and (2), the matrix $$X_i^k $$ is comprised of the following vectors:

3 $$\begin{aligned} X_i^k = Firm_i^l + Enr_i^m + Idc_i^o + Bar_i^p + Ctr_i^q + Ind_i^r \end{aligned}$$

The number of variables referred to in the superscripts on the right side sum up to *k*.

Turning to the impacts of adoption, we are interested in three dependent variables: $Imp_i^{eff}$, $Imp_i^{co2}$, and $Imp_i^{cmp}$. Reflecting the discrete ordinal nature of the dependent variables, we conduct *ordered probit* regressions with the above adoption choices entering as explanatory variables:

4 $$\begin{aligned} \begin{pmatrix} Imp_i^{eff} &{} \\ Imp_i^{co2} &{} \\ Imp_i^{cmp} &{} \\ \end{pmatrix} = \begin{pmatrix} \alpha _{10} &{} + \gamma _1^t Adp_i^{t} &{} + \beta _10^u X_i^u &{} + \upsilon _{10} \\ \alpha _{11} &{} + \gamma _2^t Adp_i^{t} &{} + \beta _11^u X_i^u &{} + \upsilon _{11} \\ \alpha _{12} &{} + \gamma _3^t Adp_i^{t} &{} + \beta _12^u X_i^u &{} + \upsilon _{12} \end{pmatrix} \end{aligned}$$

The superscript *t* denotes the adoption variables used in the impact equations. The superscript *u* denotes the general control variables. Their number must again be equal to the number of variables referred to in the superscripts on the right side of the following expression:

5 $$\begin{aligned} X_i^u = Firm_i^l + Enr_i^m + Idc_i^o + Ctr_i^q + Ind_i^r \end{aligned}$$

For all the equations, we have run manifold tests of robustness. The main relationships between our variables on policy, adoption and impacts are not sensitive to meaningful variations in the set of control variables. Similarly, using different methods of estimation, such as OLS or logit instead of probit and ordered probit models did not result in any pronounced difference. The most informative tests of the robustness of the impact equations are those using multinomial logit regressions (Tables 11, 12 and 13 in the Annex).

Finally, we want to emphasise that economics is often considered a science of ‘universal interdependence’. Endogenous relationships abound and the econometric identification of strict causal impacts can be extremely demanding. Ideally, we would therefore like to estimate the above relationships simultaneously by means of a structural equation model.^26^ But currently there has been only one wave of the enterprise survey, which implies that the available observations are not sufficient to carry such an approach.

Despite this limitation, we aim to track the presumed causal structure as closely as possible and to disentangle mutual impacts of our target variables by means of the vast array of single equations as summarised in Fig. 1. Particularly, the separation of the two sets of equations on adoption and performance is meant to reduce the possible distortions from endogenous relationships. Still one cannot preclude such interferences between the target variables. To that purpose, further research with enlarged data sets for more countries and additional waves of the enterprise survey is highly desirable. Currently, however, we must confine the interpretation of our empirical results to the establishment of stylized facts about statistically significant associations between the variables of interest.

### Results

**Fig. 2:**
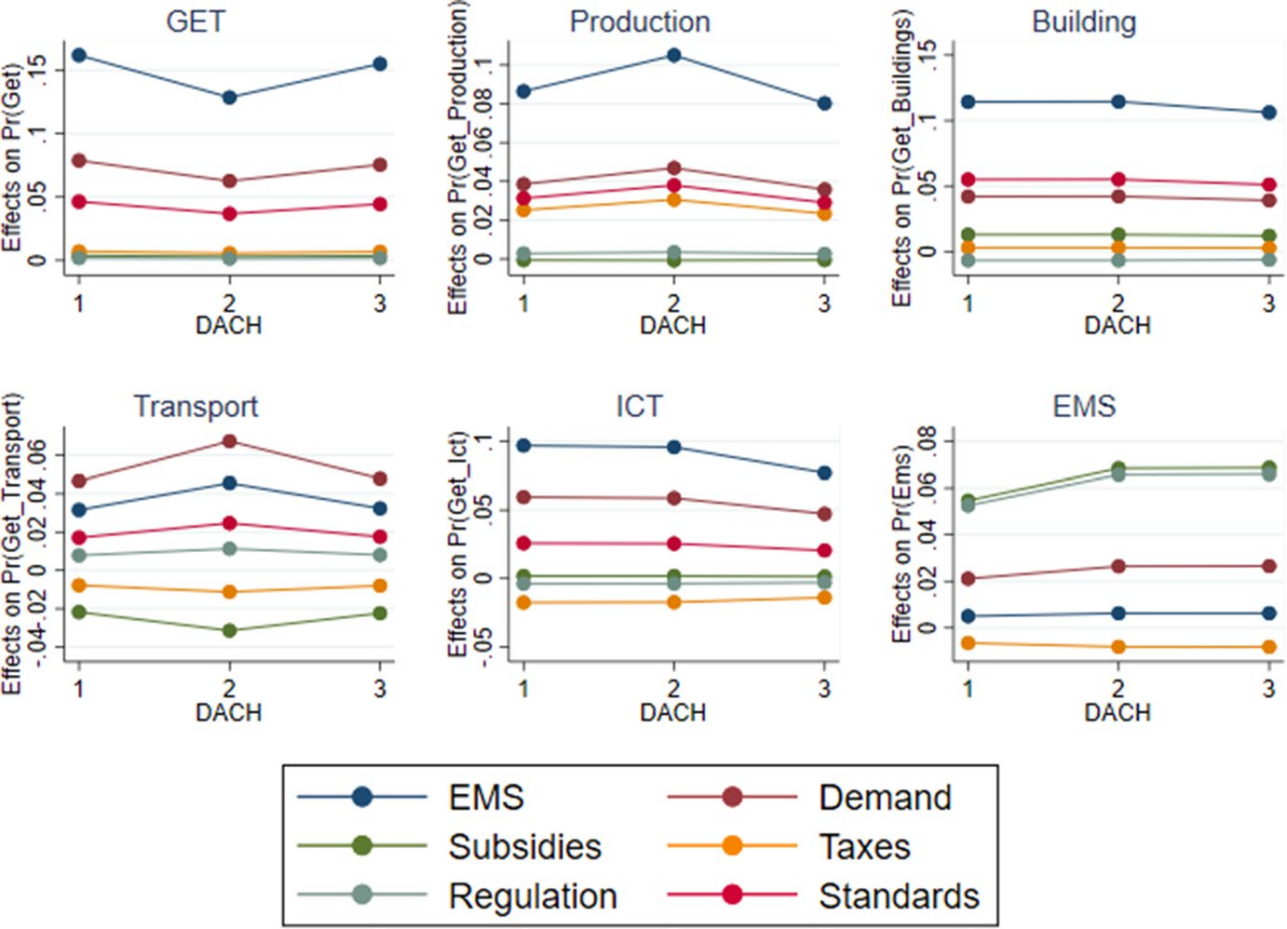
Average marginal effects (AME) on the probability of adoption. NB: Labels on the x-axis are 1 for Germany, 2 for Austria and 3 for Switzerland

**Fig. 3:**
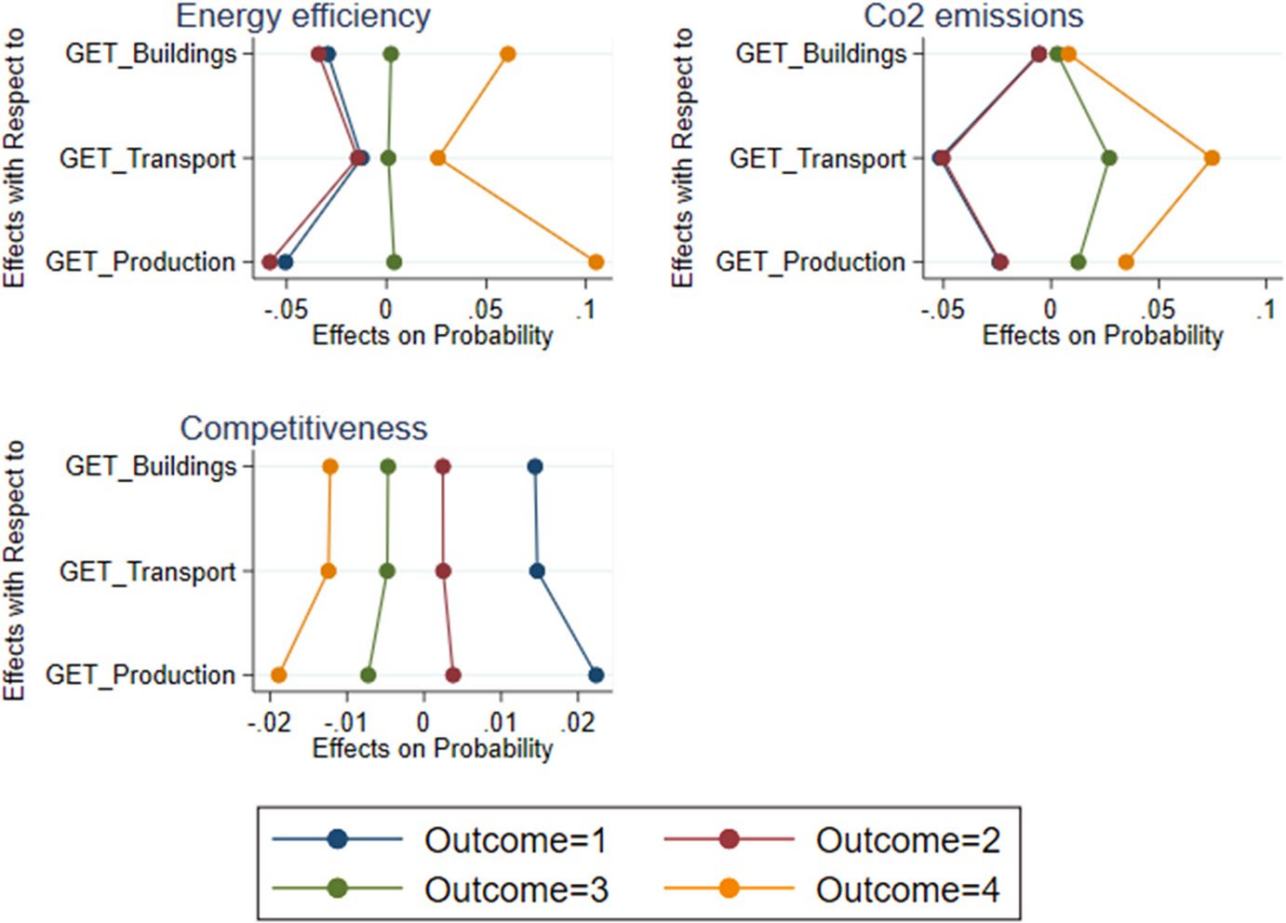
Average marginal effects (AME) on the probability of outcomes. NB: Outcomes are 1 for “not improved/worsened”, 2 for “can’t say/didn’t change”, 3 for “improved” and 4 for “much improved”

The following tables and figures summarise the results from the various econometric estimations. To begin with, Table 6 presents the coefficients of the adoption equations for EMS and GETs, while Table 7 shows them for GETs by technology field. Table 8 reports the *average marginal effect* (AME) on the respective probabilities of adoption for those factors, which are statistically significant. Figure 2 pictures the AMEs separately for German, Austrian and Swiss firms. Finally, Table 9 presents the estimates on the impact equations, followed by the AMEs for the significant adoption variables, which are on display in Table 10 and Fig. 3.

#### Adoption

Turning first to the adoption equations, the estimates reveal a strikingly differentiated picture with regard to the effectiveness of various policy instruments by technology field. For *energy-related management systems* we test how the inducement factors associate with their adoption as well as the further correlation of EMS with the adoption of GETs. In short, firms which are large, part of an enterprise group, or exporters have a higher probability of adopting EMS. In addition, environmental taxes and standards are significant policy-related factors that raise their probability of use by 6.1 and 5.9 percentage points (pp), respectively. Their introduction increases the firm’s probability of adopting new GETs, with the AME amounting to 15.7 pp overall. It is significant in each of the technological fields, but strongest in buildings, followed by ICT, production, transport and renewable energy sources. These results are thus consistent with the studies by Khanna et al. (2009) or Horbach et al. (2012), who emphasize that EMS help firms to overcome incomplete information and identify inefficiencies and opportunities for cost savings

*Customer demand* for energy-efficient products and processes is another significant driver of the adoption of new GETs in all five technology fields. The AME on the probability of adoption of firms, which report that it is (highly) relevant to their business, is 7.6 pp. It is strongest for ICT related GETs, followed by GETs in transport, production, renewable energy and buildings. Overall, this finding is consistent, for instance, with a recent study by Aghion et al. (2021). Though focusing on innovation rather than diffusion, they argue that firms seek to soften price competition by pursuing green products, if agents care about the environmental footprint of their consumption. Testing their hypothesis on a large panel of patent data in the automobile industry, they demonstrate that prosocial consumer preferences can actually ‘move markets’.^27^

**Table 6:** Explaining EMS and GET adoption

VARIABLES	Extensive margin			Intensive margin	
	EMS	GET	GET	GET	GET
EMS		0.512***	0.519***	$-$ 0.580	0.484
		(0.0695)	(0.0970)	(1.252)	(1.710)
Customer demand	0.0241	0.249***	0.204***	$-$ 0.470	$-$ 1.020
	(0.0529)	(0.0501)	(0.0678)	(0.852)	(1.154)
Public funding	0.102	0.0125	$-$ 0.0921	6.360***	5.311*
	(0.135)	(0.126)	(0.166)	(2.174)	(2.997)
Taxes	0.263***	0.0227	0.0646	0.297	$-$ 0.840
	(0.0528)	(0.0494)	(0.0713)	(0.889)	(1.250)
Regulations	$-$ 0.0314	0.00583	0.124	$-$ 5.536**	$-$ 1.792
	(0.137)	(0.130)	(0.172)	(2.228)	(3.094)
Standards	0.253***	0.147***	0.175**	$-$ 1.322	$-$ 1.932
	(0.0545)	(0.0550)	(0.0789)	(0.912)	(1.238)
Political framework	$-$ 0.00677	0.121***	0.119**	$-$ 0.330	$-$ 0.871
	(0.0434)	(0.0410)	(0.0592)	(0.680)	(0.916)
Immature tech.	$-$ 0.0305	0.136***	0.151***	$-$ 0.158	$-$ 0.224
	(0.0419)	(0.0396)	(0.0558)	(0.657)	(0.896)
Long amortisation	0.0762**	0.170***	0.178***	$-$ 1.034*	$-$ 1.084
	(0.0355)	(0.0342)	(0.0465)	(0.578)	(0.771)
Lack of finance	$-$ 0.00780	$-$ 0.140***	$-$ 0.198***	0.291	0.325
	(0.0379)	(0.0360)	(0.0510)	(0.657)	(0.901)
Energy prices	0.0436	0.0415	$-$ 0.0315	3.729***	2.993**
	(0.0520)	(0.0472)	(0.0697)	(0.870)	(1.195)
Energy mix	0.163***	0.418***	0.345***	2.304***	1.403
	(0.0415)	(0.0438)	(0.0641)	(0.649)	(0.922)
Group	0.288***	$-$ 0.0478	$-$ 0.0436	$-$ 2.414**	$-$ 1.512
	(0.0677)	(0.0636)	(0.0901)	(1.228)	(1.666)
Size class	0.593***	0.240***	0.140**	$-$ 2.931***	$-$ 1.934
	(0.0497)	(0.0484)	(0.0693)	(0.913)	(1.249)
Exports	0.397***	0.117*	0.190**	1.426	0.0239
	(0.0742)	(0.0639)	(0.0955)	(1.305)	(1.932)
Innovation (GET)			0.261**		1.102
			(0.111)		(1.862)
Austrian	0.222*	0.223*	0.100	7.803***	4.970*
	(0.117)	(0.116)	(0.163)	(1.865)	(2.628)
Swiss	0.354***	$-$ 0.529***	$-$ 0.579***	3.209**	4.036**
	(0.0742)	(0.0659)	(0.0964)	(1.344)	(1.858)
(Pseudo) $R^2$	0.293	0.219	0.219	0.157	0.147
Observations	2,923	2,959	1,442	1,282	610

Standard errors in parentheses; ***$p<0.01$; **$p<0.05$; *$p<0.1$

Included but not displayed: $Ind_i^{nace}$, $Firm_i^{age}$, $Enr_i^{cos}$, $Enr_i^{sht}$, $Bar_i^{inc}$, $Bar_i^{prm}$

**Table 7:** Explaining GET adoption by technology fields

VARIABLES	Production	Buildings	Transport	ICT	Renewables
EMS	0.440***	0.380***	0.197***	0.350***	0.227***
	(0.0675)	(0.0616)	(0.0722)	(0.0636)	(0.0790)
Customer demand	0.192***	0.149***	0.271***	0.209***	0.262***
	(0.0499)	(0.0450)	(0.0504)	(0.0444)	(0.0534)
Public funding	0.0275	0.103	$-$ 0.105	0.0424	0.120
	(0.129)	(0.114)	(0.132)	(0.115)	(0.133)
Taxes	0.108**	0.00196	$-$ 0.0401	$-$ 0.0594	0.0371
	(0.0517)	(0.0465)	(0.0538)	(0.0475)	(0.0592)
Regulations	$-$ 0.00885	$-$ 0.0569	0.0242	$-$ 0.0451	$-$ 0.133
	(0.131)	(0.116)	(0.134)	(0.118)	(0.137)
Standards	0.142***	0.190***	0.120**	0.111**	0.0987*
	(0.0518)	(0.0486)	(0.0548)	(0.0489)	(0.0596)
Political framework	0.0667*	0.0831**	0.137***	0.0459	0.0999**
	(0.0394)	(0.0364)	(0.0407)	(0.0369)	(0.0458)
Incompatible tech.	0.129***	$-$ 0.0784**	$-$ 0.0689*	0.0227	$-$ 0.110**
	(0.0377)	(0.0356)	(0.0401)	(0.0353)	(0.0458)
Immature tech.	0.0976**	0.108***	0.184***	0.0848**	0.0982**
	(0.0387)	(0.0354)	(0.0391)	(0.0355)	(0.0441)
Long amortisation	0.102***	0.183***	0.0973***	0.0882***	0.0242
	(0.0326)	(0.0298)	(0.0342)	(0.0304)	(0.0385)
Lack of finance	$-$ 0.101***	$-$ 0.105***	$-$ 0.106***	0.0420	$-$ 0.102**
	(0.0355)	(0.0325)	(0.0377)	(0.0327)	(0.0433)
Energy prices	0.0972*	0.0196	0.100*	0.0112	$-$ 0.0121
	(0.0509)	(0.0444)	(0.0517)	(0.0456)	(0.0584)
Energy shortage	0.0250	$-$ 0.0297	$-$ 0.0248	0.114**	$-$ 0.0339
	(0.0528)	(0.0480)	(0.0550)	(0.0478)	(0.0623)
Energy mix	0.156***	0.331***	0.133***	0.153***	0.513***
	(0.0395)	(0.0358)	(0.0402)	(0.0360)	(0.0392)
Age	0.00124*	0.00177***	0.00177**	$-$ 0.000886	0.00133*
	(0.000729)	(0.000636)	(0.000729)	(0.000692)	(0.000799)
Size class	0.294***	0.276***	0.208***	0.169***	0.0154
	(0.0475)	(0.0417)	(0.0482)	(0.0427)	(0.0542)
Austrian	0.169	0.266***	0.270***	$-$ 0.175*	0.421***
	(0.105)	(0.0929)	(0.104)	(0.0945)	(0.110)
Swiss	$-$ 0.310***	$-$ 0.419***	$-$ 0.109	$-$ 0.402***	$-$ 0.102
	(0.0747)	(0.0632)	(0.0750)	(0.0644)	(0.0847)
Pseudo $R^2$	0.301	0.224	0.203	0.119	0.199
Observations	3,369	3,369	3,369	3,369	3,369

Standard errors in parentheses; ***$p<0.01$; **$p<0.05$; *$p<0.1$. Pseudo $R^2$ is calculated for single equations

Included but not displayed: $Ind_i^{nace}$, $Enr_i^{cost}$

Energy-related *standards* and negotiated agreements are the third persistent driver of the extensive margin of adoption for GETs in all five technology fields.^28^ The AME on the adoption of any GET is 5.9 pp. It is highest for buildings, followed by production, ICT, transport and renewable energy. Furthermore, standards also exert an indirect impact on the introduction of GETs by raising the probability of adopting EMS by 5.9 pp.

Such an indirect impact appears to be the most significant influence of energy-related *taxes and duties*, where the AME on the introduction of EMS amounts to 6.1 pp. In contrast, the estimates show no significant direct impact on the adoption of GETs in any technology field, except production (with an AME of 2.5 pp). This result is largely consistent, for instance, with the findings of Lanoie et al. (2011), who report a significant impact on environmental R&D and self-reported environmental performance only when environmental taxes are perceived to be very high (which they argue is not very common among OECD countries).

While neither of the above instruments appears to have a significant impact on the share of GETs in total investments, *public funding* and regulation (other than standards) only affect this intensive margin of adoption. Public funding does so consistently if we either include or leave out own innovations in GETs from the set of control variables. In contrast, the findings on *regulations* (other than standards) are inconclusive as their impact is negative in one specification, but becomes insignificant if we include own innovations among the explanatory variables in the adoption of GETs.^29^

Overall, the heterogenous picture which emerges from our estimations on the adoption equations is consistent with the received empirical literature. For instance, the survey on the diffusion of green technologies by Allen et al. (2013) concludes that both market-based and regulatory instruments can be effective depending on the particular situation. They point, among others, at Gallagher and Muehlegger (2011), who demonstrated the effectiveness of tax incentives for the rate of adoption of hybrid vehicles, or at the influential study by Popp (2010), which identified environmental regulations to be the key driver for the adoption of pollution control technologies.^30^

**Table 8:** Average marginal effects (AME) on the probability to adopt (in pp)

Inducement	Adoption						
	$Adp_i^{ems}$	$Adp_i^{get}$	$Adp_i^{prd}$	$Adp_i^{bld}$	$Adp_i^{trp}$	$Adp_i^{ict}$	$Adp_i^{ren}$
$Adp_i^{ems}$	n.a.	15.7***	8.6***	11.1***	3.3**	8.8***	3.0**
DE	n.a.	16.2***	8.7***	11.4***	3.2**	9.7***	2.8**
AT	n.a.	12.9***	10.5***	11.5***	4.6**	9.6***	5.1**
CH	n.a.	15.5***	8.0***	10.6***	3.2**	7.7***	2.8**
$Idc_i^{dem}$	–	7.6***	4.1***	4.1***	4.9***	5.4***	4.0***
DE	–	7.9***	4.2***	3.9***	4.7***	5.9***	3.8***
AT	–	6.3***	4.2***	4.7***	6.7***	5.8***	6.8***
CH	–	7.6***	3.9***	3.6***	4.8***	4.7***	3.7***
$Idc_i^{std}$	5.9***	5.5***	3.1***	5.3***	1.8*	2.4*	–
DE	5.3***	4.6***	3.1***	5.5***	1.7*	2.6*	–
AT	6.6***	3.7***	3.8***	5.5***	2.5*	2.6*	–
CH	6.6***	4.5***	2.9***	5.1***	1.8*	2.1*	–
$Idc_i^{tax}$	6.1***	–	2.5**	–	–	–	–
DE	5.5***	–	2.5**	–	–	–	–
AT	6.8***	–	3.1**	–	–	–	–
CH	6.9***	–	2.3**	–	–	–	–

Marginal effects displayed only if statistically significant: ***$p<0.01$; **$p<0.05$; *$p<0.1$

Turning to the potential barriers to the introduction of new GETs, the *lack of finance* is perceived to be a significant impediment to adoption. This applies to all technology fields, except ICT.^31^ High and volatile *energy prices* significantly raise the probability of GET adoption only in production and transport, but generally associate with a higher intensive margin. Fears of *energy shortages* only affect the extensive margin in the field of ICT. Rather than acting as substitutes, effective changes in the energy mix of the firm complement the adoption of EMS and new GETs.

Adding the variable on own *innovation* in the field of energy-related technologies to the set of regressors reduces the sample by more than one half. We therefore display the outcome in two separate columns of Table 6. This also serves as a test of the robustness with respect to smaller sample sizes. While the other findings remain unaffected, own innovations with regard to GETs have a significant and positive impact on the extensive margin but not on the intensive margin. This aligns with recent findings by Van Leeuwen and Mohnen (2017), who show that eco-investment and eco-innovation are complementary.

Among other firm characteristics, *group* membership relates to a lower intensive margin of GETs. Firm *size* by number of employees generally associates with a higher extensive margin (except for renewable energy). And so does *age* (except if applied to ICTs). In comparison to German enterprises, Austrian firms more often report the adoption of EMS and GETs (except for ICT) and also exhibit a higher share of GETs in total investment. Compared to German firms, Swiss enterprises show a higher probability of introducing EMS, but a lower extensive margin and a higher intensive margin of adopting GETs. But when comparing the AMEs in Table 8 and Fig. 2, the systematic differences between policy instruments clearly dominate any differences between the three countries of the DACH region.

#### Impacts

The second set of equations is directed at the perceived impacts of adopting EMS and GETs on energy efficiency, $\hbox {CO}_{2}$ emissions, and the firm’s competitiveness. Different activities condition the *genuine objective* of adoption. On the one hand, firms that have introduced an EMS show a significantly higher propensity to adopt new GETs with the primary objective of raising energy efficiency or reducing $\hbox {CO}_{2}$ emissions (see the first column in Table 9). On the other hand, the propensity also rises with the importance of customer demand for energy efficient products and services, the overall intensive margin of adoption, and the extensive margin in the area of buildings. For firms that have expressed a concern about energy shortages, the ecological impacts are more often only a secondary effect. Not surprisingly, the genuine purpose of energy savings improves the ecological impacts of adoption, which implies that mere windfalls from promoting general investments and the modernisation of facilities won’t achieve an equivalent outcome.

**Table 9:** Objectives and impacts of GET adoption

VARIABLES	Objective	Energy efficiency	Carbon emissions	Competitiveness
EMS	0.352***	0.0707	0.154*	0.00863
	(0.0833)	(0.0796)	(0.0789)	(0.0805)
Objective		0.175***	0.123***	0.0298
		(0.0433)	(0.0428)	(0.0434)
Adoption: Production	$-$ 0.0536	0.343***	0.128*	$-$ 0.0863
	(0.0818)	(0.0777)	(0.0768)	(0.0784)
Adoption: Buildings	0.261***	0.199***	0.0298	$-$ 0.0558
	(0.0791)	(0.0737)	(0.0737)	(0.0750)
Adoption: Transport	0.0453	0.0847	0.276***	$-$ 0.0568
	(0.0817)	(0.0781)	(0.0772)	(0.0784)
Adoption intensity	0.0100***	0.00438**	0.00434**	$-$ 0.000951
	(0.00194)	(0.00188)	(0.00185)	(0.00187)
Energy cost 2012	0.00766	$-$ 0.00719**	$-$ 0.00393	0.000856
	(0.00558)	(0.00336)	(0.00343)	(0.00338)
Energy prices	0.00575	0.130***	0.0460	$-$ 0.0928*
	(0.0584)	(0.0500)	(0.0495)	(0.0505)
Energy shortage	$-$ 0.135**	$-$ 0.0315	0.0572	0.0340
	(0.0611)	(0.0561)	(0.0558)	(0.0565)
Group	$-$ 0.0837	$-$ 0.186**	$-$ 0.127	0.0849
	(0.0832)	(0.0782)	(0.0778)	(0.0792)
Competition	$-$ 0.0247	$-$ 0.0680***	$-$ 0.0187	$-$ 0.0142
	(0.0253)	(0.0237)	(0.0236)	(0.0240)
Customer demand	0.189***			
	(0.0568)			
Austrian	0.270**	0.271**	0.204*	0.990***
	(0.122)	(0.114)	(0.114)	(0.116)
Swiss	$-$ 0.116	0.790***	0.237***	1.592***
	(0.0873)	(0.0846)	(0.0815)	(0.0875)
Pseudo $R^2$	0.091	0.080	0.051	0.156
Observations	1,245	1,234	1,217	1,232

Standard errors in parentheses; ***$p<0.01$; **$p<0.05$; *$p<0.1$.

Included but not displayed: $Ind_i^{nace}$, $Firm_i^{sze}$, $Firm_i^{exp}$; only column 1: $Idc_i^{pfu}$, $Idc_i^{tax}$, $Idc_i^{reg}$, $Idc_i^{std}$

**Table 10:** AME on probability that ...“much improved” (in pp)

Adoption of GETs	Impacts	
	*Energy efficiency*	$\hbox {CO}_{2}$ *emissions*
*Production*	10.5***	3.5*
DE	9.3***	3.2*
AT	11.3***	4.1*
CH	12.5***	3.9*
*Transport*	–	7.5***
DE	–	6.8***
AT	–	8.9***
CH	–	8.3***
*Buildings*	6.1***	–
DE	5.4***	–
AT	6.5***	–
CH	7.2***	–

Marginal effects displayed only if statistically significant: ***$p<0.01$; **$p<0.05$; *$p<0.1$

The self-reported ecological impacts of adopting new GETs differ considerably by technological fields. For example, new GETs in *production* associate significantly with higher energy efficiency and reduced $\hbox {CO}_{2}$ emissions. Compared to the adoption of GETs other than in production, the AME on the self-reported assessment that either has much improved is 10.5 pp and 3.5 pp, respectively (Table 10). For energy efficiency, the AME ranges from 12.5 pp in Switzerland to 11.3 pp in Austria and 9.3 pp in Germany. Regarding $\hbox {CO}_{2}$ emissions, the range is much smaller (from 4.1 pp in Austria, followed by 3.9 pp in Switzerland and 3.2 pp in Germany).

In *transport* the adoption of GETs is perceived to have significantly reduced the firm’s $\hbox {CO}_{2}$ emissions without significantly enhancing its energy efficiency. For the self-reported assessment that $\hbox {CO}_{2}$ emissions have much improved, the AME of the full sample is 7.5 pp. It is highest in Austria, followed by Switzerland and Germany. Conversely, the adoption of new GETs in *buildings* significantly improves energy efficiency, but not the $\hbox {CO}_{2}$ imprint of the adopting firm. The AME on the self-reported assessment that energy efficiency has much improved is 6.1 pp. Here Switzerland has the highest AME, followed by Austria and Germany.

Turning to the impact of new GETs on the firm’s competitiveness, Cohen and Tubb (2018) report considerable heterogeneity among the observed impacts of environmental regulations on economic performance. In their meta-analysis of 103 studies, positive and negative effects are about equally likely, while the most frequent outcome is that of a statistically insignificant relationship. Different from our study, however, the focus is generally on environmental innovations. Overall, Cohen and Tubb (2018) conclude that positive effects are more likely with studies at the level of countries or regions, whereas negative effects are more likely observed at the level of facilities, firms or industries.^32^

While these findings from the literature on firm-level innovations may lead one to expect a negative sign, our findings support the hypothesis of a largely neutral effect of the adoption of new GETs on the competitiveness of the average firm. This result points towards the general fact that the need and incentives for adoption apply similarly to firms in the same market, leaving little scope for differential impacts on their relative competitive position. Furthermore, the potential surplus of early adoption would only apply to a few firms and not significantly affect the average enterprise in the sample. Overall, our results are thereby consistent with Van Leeuwen and Mohnen (2017), who report that subsidies, energy price incentives and environmental regulations affect environmental investments (though in particular eco innovations), but show no significant impact on total factor productivity.

Finally, among the auxiliary factors, group membership and more intense competition appear to reduce the economically feasible options in adopting GETs, while significantly decreasing their impact on energy efficiency. Using enterprise surveys one must, however, generally stay alert to the subjective nature of the data. For example, if respondents expect that their self-reported assessments may influence future policies, the relevance of more restrictive and disliked policies may be biased downwards in comparison to weaker interventions. Conversely, the assessment of policies with a direct pecuniary benefit to the firm may be biased upwards. The low and insignificant impact of subsidies on the extensive margin of adoption may, however, indicate that the particular design of the survey helped to mitigate such a bias (see Sect. 3.1).

## Policy conclusions

Time is running short and the United Nations (2020) has urgently called for a Decade of Action to achieve the ambitious Sustainable Development Goals (SDGs) by the year 2030. Even if these prove technologically feasible, their implementation will remain an arduous political task. One major obstacle is the widespread fear that mandatory environmental restrictions negatively affect the competitiveness of firms to which they apply. Exploiting the micro-data of a new enterprise survey for Germany, Austria and Switzerland, we tested how different environmental policy instruments relate to the adoption of “green” energy technologies (GETs) and to the self-reported ecological and economic performance of individual firms. The analysis offers several conclusions for economic policy:

1. **Policy agenda.** At a fundamental theoretical level, public interventions to foster GETs aim to increase a system’s access to free energy and to raise the efficiency in its use. With regard to the perils of climate change, the very long-time horizon, presumed nonlinear effects, and the risk of irreversible catastrophic events call for a *conservationist* bias.
2. **Policy instruments.** Conventional analyses tend to prefer incentive-based tools, which minimize the costs of an intervention for a given ecological impact, over command-and-control type of interventions. Alternative approaches emphasize institutional complexity, where e.g. standards and other regulations shape the selection environment to which firms must adapt. Our empirical results call for a comprehensive combination of both. They demonstrate that policy associates with the adoption of GETs, but not uniformly and via *differentiated channels of transmission*:
  - Customer demand is a major inducement factor, which points at *education* and *public awareness* as important policy tools to shape what society accepts as sustainable methods of production.
  - *Standards and negotiated agreements* as well as energy related management systems (EMS) appear to be among the most consistent drivers of adoption. These findings point at the high practical relevance of detailed technical rules.
  - Energy-related *taxes* and duties show a significant association only in the field of production and with the adoption of EMS. Their limited impact, however, may also indicate insufficient use of incentive-based tools in the DACH region rather than a lack of effectiveness *per se*.
  - In our sample, *public funding* shows no significant association with the *extensive* margin of adoption. This may indicate that in practice most funding institutions neither have the information to identify, which firms are at the margin of adoption, nor the means to restrict their support accordingly.
  - Still, the significant coefficient on the *intensive margin* suggests a positive role of public funding in the adoption of GETs. One likely reason is that it affects choices within a company in favour of activities that earn the subsidy and thereby help to increase their budgets relative to others.
  Comparing the three countries in the DACH region, Austrian companies report the highest adoption rate for GETs and EMS. German companies are second in terms of the extensive margin of GET adoption, but behind companies from Switzerland in terms of the intensive margin of GET adoption.
3. **Policy impacts.** Public debates typically center on two opposing hypotheses. One says that the conversion to green energy would harm competitiveness because it engenders additional costs. Another view, known as the Porter hypothesis, maintains that companies that implement stricter regulations and stricter environmental rules faster will be rewarded by a competitive edge. Essentially, we found that success lies primarily on the ecological side:
  - According to our survey data the overall adoption of GETs significantly improves *energy efficiency* and reduces $\hbox {CO}_{2}$ emissions. The above policies to foster GET adoption are therefore largely consistent with the environmental objectives among the United Nations’ goals for sustainable development.
  - The average marginal effect (AME) of GET adoption on energy efficiency tends to be highest in Switzerland, followed by Austria, while Germany ranks third. For the AME on $\hbox {CO}_{2}$ emissions, Austrian firms lead by a small margin over Switzerland, followed by Germany.
  - Finally, the firms’ *competitiveness* was neither measurably impaired nor enhanced by the introduction of new GETs. Contrary to the aforementioned hypotheses, the overall effect is neutral. This result strengthens the case for public policies that promote the needed energy transition without necessarily compromising the United Nations’ goals for growth and employment.

Several reasons can explain the latter result in support of the *neutrality hypothesis*. To begin with, energy cost is a substantial but not major share of total costs in most of the companies. Moreover, energy efficiency also leads to cost savings for the respective companies. Finally, many firms, especially in the services sector, operate and compete locally. In that case, for instance, an increase in the price of electricity affects all competing firms in the same way, meaning that none of them suffers a differential disadvantage for comparable levels of energy intensity in production.

As a general caveat, however, it must also be stressed that the econometric estimates are strictly for the average firm in our sample, which covers many different industries. Therefore, there may well be competitive disadvantages for companies in energy-intensive sectors such as the steel industry or the transport sector, wherever the respective companies are additionally exposed to strong international competition. While the costs caused by the development and introduction of new GETs are often cushioned by public funding, many environmental regulations provide exemptions for energy intensive companies to deliberately cater for this situation. Such measures highlight the persisting trade-offs between environmental goals and competitiveness concerns. Their resolution will require additional instruments, such as the introduction of carbon border adjustments (CBAs) in order to reconcile the goals of curbing carbon leakage with those of a comprehensive pricing of $\hbox {CO}_{2}$ emissions.

For the average firm, however, our analysis has shown that environmental policies significantly relate to higher energy efficiency and an improved carbon footprint, without having a significant self-reported negative impact on its competitiveness. Moreover, the analysis has shown how different instruments capitalize on distinct strengths and opportunities, demonstrating the need for a comprehensive policy mix that aims to induce the adoption of GETs directly by means of technical standards, energy related taxes or subsidies, and more indirectly, for instance, by targeting demand or the implementation of EMS.

## Annex: Supplementary tables

See Tables 11, 12 and 13.

**Table 11:** Impact of GETs on energy efficiency - MLogit

VARIABLES	Not improved	Improved	Much improved
	*vs* ‘Can’t say’		
Adoption: Production	0.201	0.780***	0.932***
	(0.299)	(0.202)	(0.227)
Adoption: Buildings	$-$ 0.437	0.131	0.331
	(0.269)	(0.186)	(0.220)
Adoption: Transport	$-$ 0.344	0.155	$-$ 0.00995
	(0.310)	(0.204)	(0.231)
Adoption intensity	$-$ 0.00577	$-$ 0.000325	0.00893*
	(0.00858)	(0.00503)	(0.00537)
Objective	$-$ 0.0598	$-$ 0.0533	0.543***
	(0.175)	(0.112)	(0.127)
EMS	$-$ 0.175	$-$ 0.00286	0.123
	(0.310)	(0.208)	(0.227)
Energy prices	$-$ 0.137	0.206	0.270*
	(0.194)	(0.130)	(0.146)
Energy supply	0.211	$-$ 0.0906	0.0792
	(0.213)	(0.149)	(0.167)
Energy cost 2012	$-$ 0.0120	$-$ 0.0209	$-$ 0.0206*
	(0.0166)	(0.0155)	(0.0120)
Group	0.159	$-$ 0.571***	$-$ 0.403*
	(0.290)	(0.202)	(0.228)
Size class	$-$ 0.0216	0.154	$-$ 0.0252
	(0.221)	(0.147)	(0.168)
Competition	0.157*	$-$ 0.0201	$-$ 0.117*
	(0.0913)	(0.0599)	(0.0705)
Exports	$-$ 0.350	$-$ 0.174	$-$ 0.270
	(0.312)	(0.216)	(0.242)
Austrian	$-$ 1.141	0.633**	0.694*
	(0.784)	(0.287)	(0.354)
Swiss	1.287***	$-$ 0.967***	2.662***
	(0.309)	(0.274)	(0.252)
Observations	1,234	1,234	1,234

Standard errors in parentheses; ***$p<0.01$; **$p<0.05$; *$p<0.1$

**Table 12:** Impact of GETs on $\hbox {CO}_{2}$ emissions - MLogit

VARIABLES	Not improved	Improved	Much improved
	*vs* ‘Can’t say’		
Adoption: Production	0.0355	0.481**	0.184
	(0.257)	(0.191)	(0.216)
Adoption: Buildings	$-$ 0.448*	$-$ 0.0956	$-$ 0.290
	(0.245)	(0.185)	(0.212)
Adoption: Transport	$-$ 0.556*	0.461**	0.292
	(0.287)	(0.192)	(0.215)
Adoption intensity	$-$ 0.00385	$-$ 0.00524	0.0112**
	(0.00693)	(0.00486)	(0.00481)
Objective	$-$ 0.0832	0.0677	0.300**
	(0.153)	(0.106)	(0.123)
EMS	$-$ 0.129	$-$ 0.200	0.424*
	(0.266)	(0.200)	(0.219)
Energy prices	0.0647	0.295**	0.0689
	(0.168)	(0.125)	(0.140)
Energy supply	0.112	0.0432	0.271*
	(0.194)	(0.141)	(0.156)
Energy cost 2012	$-$ 0.00363	$-$ 0.00669	$-$ 0.0122
	(0.0115)	(0.0110)	(0.00984)
Group	0.496*	$-$ 0.143	$-$ 0.0128
	(0.257)	(0.197)	(0.222)
Size class	0.0276	0.0924	$-$ 0.0395
	(0.193)	(0.138)	(0.160)
Competition	0.0646	0.0135	$-$ 0.0138
	(0.0809)	(0.0583)	(0.0689)
Exports	$-$ 0.307	0.245	$-$ 0.0206
	(0.268)	(0.214)	(0.235)
Austrian	$-$ 0.252	0.663**	0.832**
	(0.580)	(0.262)	(0.338)
Swiss	2.241***	$-$ 0.736***	2.455***
	(0.264)	(0.264)	(0.231)
Observations	1,217	1,217	1,217

Standard errors in parentheses; ***$p<0.01$; **$p<0.05$; *$p<0.1$

**Table 13:** Motive for adopting GETs - MLogit

VARIABLES	Both objectives	Primary motive
	*vs* Secondary effect	
Adoption: Production	0.249	$-$ 0.193
	(0.173)	(0.197)
Adoption: Buildings	0.170	0.643***
	(0.163)	(0.198)
Adoption: Transport	0.264	0.0462
	(0.173)	(0.200)
Adoption intensity	0.0136***	0.0236***
	(0.00452)	(0.00475)
EMS	0.627***	0.780***
	(0.177)	(0.202)
Customer demand	0.216*	0.479***
	(0.124)	(0.138)
Public funding	0.260	0.353
	(0.326)	(0.357)
Taxes	$-$ 0.119	0.176
	(0.127)	(0.143)
Regulations	0.00709	$-$ 0.108
	(0.333)	(0.366)
Standards	0.0743	0.132
	(0.130)	(0.145)
Energy cost 2012	0.00240	0.0170
	(0.0150)	(0.0147)
Energy prices	0.218*	$-$ 0.0331
	(0.124)	(0.141)
Energy supply	0.0989	$-$ 0.397**
	(0.127)	(0.160)
Group	$-$ 0.195	$-$ 0.177
	(0.175)	(0.202)
Size class	0.102	0.108
	(0.126)	(0.143)
Austrian	0.245	0.624**
	(0.275)	(0.286)
Swiss	$-$ 0.136	$-$ 0.292
	(0.184)	(0.216)
Observations	1,245	1,245

NB: Standard errors in parentheses; ***$p<0.01$; **$p<0.05$; *$p<0.1$

Included but not displayed: fixed industry effects; competition and exports

## Abbreviations

AME: Average Marginal Effect
DACH: Germany (D), Austria (A) and Switzerland (CH)
EMS: Energy Management System
GET: Green Energy Technology

## Footnotes

1 United Nations (2020, p. 2).

2 “Climate change has very unequal impacts: it is usually the poorest people who are hit earliest and hardest; they live in more vulnerable areas, are less-well insured, and have weaker coping mechanisms” (Stern and Stiglitz (2021, p. 5).

3 Millner and Ollivier (2016).

4 Arrow and Fisher (1974), Weitzman (1998), Pindyck (2007).

5 See Jaffe et al. (2005), Popp et al. (2010) or Popp (2019). The negative external effects result from distorted price signals (Pigou 1920) or incomplete property rights (Coase 1960). Stavins (2011) discusses both as a problem of the commons. Ostrom (1990, 2010) demonstrates the possibility of local communities to contain these by means of self-organized rules and institutional arrangements. However, for climate change—the ‘ultimate commons problem’—the community affected is global and potential barriers for coordination and governance are immensely more difficult.

6 van den Bergh (2007), Gillingham and Palmer (2014), Peneder (2017).

7 On the “environmental Kuznets curve” see, e.g., Grossman and Krueger (1995), Fouquet (2014), Moosa (2017) or Halkos and Managi (2017).

8 Georgescu-Roegen (1971), Ayres (1994), Buenstorf (2000), van den Bergh (2007), or Foster (2014).

9 Lehr and Lutz (2016) and Rammer et al. (2017).

10 They were introduced by the Electricity-Feed-In Act of 1991, which was succeeded by the Renewable Energy Act (EEG) in 2000 and repeatedly revised since.

11 AGEB (2017).

12 Apart from valid concerns about carbon leakage, this institutional setting contributed, for instance, to a general ceiling of energy levies to a maximum of 0.5% of a company’s total value added. See Kletzan-Slamanig and Köppl (2016) or Köppl and Schratzenstaller (2015).

13 Missing the target triggers a penalty, which is paid to a fund that promotes energy-saving activities. While energy using firms are not directly charted for missing the target, they are allowed to sell their efficiency measures to their energy supplier. Similarly, there is no regulatory protection against suppliers discriminating prices to the disadvantage of firms that cannot offer such savings.

14 Stucki and Wörter (2016).

15 The distinction between an *extensive* and an *intensive* margin is particularly useful in the study of survey data, when a variable has many zero entries. For example, in international economics the extensive margin regularly depicts whether a firm is exporting or not, while the intensive margin reports the ratio of exports to total sales. Similarly, in innovation studies the extensive margin tells, whether a firm does any own R&D, whereas the intensive margin reports the ratio of R&D expenditures to its total sales.

16 The adoption of energy-saving technologies is a special case, since it also reduces expenditures on current operations. This effect, however, has already been covered by the above hypothesis on energy efficiency. When we address the impact of adoption on *competitiveness* proper, we ask differently for the specific impact of the new technology on the firms’ relative position to its main competitors in the market.

17 While the most compelling part of the Porter hypothesis relates regulation to incentives for own innovation, the meaning of innovation is not exactly specified in their analysis. For example, when Porter and van der Linde refer to the benefits of regulatory signals with respect to resource inefficiencies, potential technological improvements, or the reduction of uncertainty for investments, the argument apparently encompasses the case of adopting new environmentally friendly technologies.

18 André et al. (2009), Constantatos and Herrmann (2011) and Ambec et al. (2013).

19 Named after psychologist Rensis Likert (1903–1981), respondents are asked to rate their level of agreement or disagreement with a series of statements on a symmetric scale (in this case comprised of three possible answers). See, e.g., Lanoie et al. (2011) or Stucki and Wörter (2016) for similar approaches.

20 See Rammer et al. (2017) for a discussion and further references.

21 In the period covered, the European $\hbox {CO}_{2}$ trading scheme had little impact on the energy costs of firms, owing to the abundance of $\hbox {CO}_{2}$ certificates and the resulting low price for $\hbox {CO}_{2}$ emissions rights (Joltreau and Sommerfeld 2016).

22 Arvanitis et al. (2016) offer further details on the comparisons between the three DACH countries.

23 Broadly defined as manufacturing, energy supply, water supply, and waste management.

24 Because of the small number of relevant observations, we ignore this variable in the later econometric analysis.

25 Capellari and Jenkins (2003).

26 As done, for instance, for different purposes and with different data in Peneder and Woerter (2014), Friesenbichler and Peneder (2016), or Peneder et al. (2019).

27 Provided that they are leveraged by effective competition between firms.

28 Note that standards are slightly above the margin of significance in the preferred multivariate probit estimation and slightly below it when using bivariate probits for the calculation of AMEs. For otherwise identical specifications and coefficients, the difference in significance is due to the higher efficiency of the multivariate technique.

29 This may point at failures in the design and execution of specific initiatives (such as the EU emission trading or the Austrian energy efficiency law; see Sect. 1). However, it may also relate to difficulties in the design of the enterprise survey, where this category was meant to capture a variety of heterogenous regulatory interventions (other than standards). Clearly, the inconclusive outcome on this variable suggests a more detailed enumeration of specific initiatives if future surveys become possible.

30 Similarly, more recent findings show the benefits of a differentiated policy mix on firms’ own innovation activity, mapped for example by patents in biofuels and housing (Costantini et al. 2015, 2017) or R&D expenditures for renewable energy (Rogge and Schleich 2018).

31 Some suspected barriers of adoption show a significant but positive statistical association with the extensive margin. This is a well-known problem with survey data and reflects the greater awareness that adopting firms have of the respective barriers. In the preferred specification we keep them as control variables, but also test the sensitivity of results when removing them. None of the coefficients was affected in a relevant manner, except that the lack of finance then became insignificant.

32 For example, Marin and Lotti (2017) report lower effects on labour productivity of eco-innovations (measured by patents) compared to other innovations.
